# Myxofibrosarcoma of the penis in an African pygmy hedgehog (*Atelerix albiventris*) – A clinical case

**DOI:** 10.17221/107/2023-VETMED

**Published:** 2024-04-25

**Authors:** Lucia Kasalova, Hana Cernochova, Radka Dvorakova, Aneta Angelova, Zdenek Knotek

**Affiliations:** ^1^Avian and Exotic Animal Clinic, Faculty of Veterinary Medicine, University of Veterinary Sciences Brno, Brno, Czech Republic; ^2^Department of Imaging Methods, Faculty of Veterinary Medicine, Dog and Cat Clinic, University of Veterinary Sciences Brno, Brno, Czech Republic; ^3^Department of Pathological Morphology and Parasitology, Faculty of Veterinary Medicine, University of Veterinary Sciences Brno, Brno, Czech Republic

**Keywords:** neoplasia, reproductive tract, small mammals

## Abstract

A 3-year-old, 420 g, intact male African pygmy hedgehog (*Atelerix albiventris*) was presented with a sudden appearance of a mass protruding from its preputium. A detailed physical examination revealed the presence of a polyp-like mass, connected to the mucous membrane of the penis and a second, multilobular mass with a larger base. Both masses were surgically removed. While the histopathological examination of the polyp-like mass revealed only a chronic active inflammatory reaction, the histopathological examination of the multilobular mass revealed a tumorous tissue composed of spindle-shaped cells, irregularly oval or polygonal in some places. Focal tumour cells with a myxoid differentiation were observed in the greater part of this tumour. The stroma was made up of sparse fibrous tissue. The surface epithelium was hyperplastic with ulcerations and necrosis. The tumour was classified as a myxofibrosarcoma. Two weeks post-surgery, the patient did not show any clinical signs of the presented disease. According to our knowledge, this is the first published case of the surgical treatment of penile myxofibrosarcoma in an African pygmy hedgehog.

## Case presentation

Up to date, a limited amount of information is available regarding the physiology and pathology of the reproductive tract in male African pygmy hedgehogs. Most published cases reported diseases associated only with the female reproductive tract (Mikaelian and Reavill 2004; Chamber et al. 2018; Okada et al. 2018; Efendic et al. 2019). Recently, one study in a male African pygmy hedgehog described a myxoma as a benign penile tumour (Takami et al. 2017). The aim of this article is to present the case of the surgical treatment of a penile myxofibrosarcoma in an African pygmy hedgehog.

**Figure 1:**
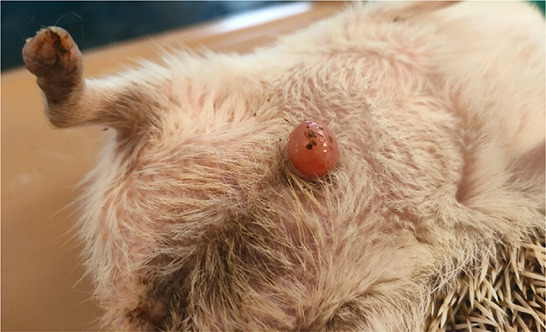
African pygmy hedgehog, male, 3-year-old Protruding polyp-shaped mass from the preputium

A 3-year-old, 420 g, intact male African pygmy hedgehog (*Atelerix albiventris*) was presented with a sudden appearance of a mass protruding from its preputium (Figure 1). It showed no other signs of distress nor any disease symptoms. The owner stated the patient was able to urinate and defecate normally at least 12 h prior to the mass appearance, although the owner did not observe any fresh urine after the mass appeared. A physical examination of the patient revealed the presence of a polyp-like mass connected to the mucous membrane of the penis. The size of the polyp-like mass was 1 × 1 cm. Palpation detected additional swelling of the lower abdominal region around the penis with another soft mass which did not allow extruding the penis from the prepuce for a complete examination. No foreign materials like hair or fur were visible and trapped in the sheath of the penis. The differential diagnosis consisted of the prolapse of the penis, posthitis of the preputium and a penile mass.

With the owner’s consent, standard diagnostic methods were used. The animal was induced to a short-lasting general anaesthesia using 3% isoflurane (Aerrane 100% 250 ml; Baxter S.A.Bd., Lessines, Belgium) concentration with a 2 l/min O_2_ flow. This allowed us to obtain a blood sample from the cranial *vena cava*. The blood plasma biochemistry analysis (performed by a DPC Konelab 20i biochemical analyser; ThermoFisher Scientific, Waltham, USA) did not reveal any abnormal values (Table 1). A haematological examination was also performed with an analyser (Celltac alpha MEK 6318 haematological analyser; Nihon Kohden, Tokyo, Japan). This revealed that the leucocyte number was within the reference interval (12.53 × 10^9^/l) with monocytosis (1.04 × 10^9^/l) (Table 2).

**Table 1:** Plasma biochemistry profile of the male African pygmy hedgehog

Parameter	SI units	Value	RI (Ivey and Carpenter 2020)
Protein	g/l	56.0	58 ± 7
Albumin	g/l	26.3	29 ± 4
Bilirubin	μmol/l	< 0.9	5.13 ± 5.13
Creatinine	μmol/l	20.1	35.37 ± 17.68
Urea	mmol/l	7.6	10.68 ± 3.21
Glucose	mmol/l	5.9	4.94 ± 1.67
ALP	μkat/l	0.49	0.85 ± 0.35
ALT	μkat/l	0.48	0.88 ± 0.4
AST	μkat/l	0.53	0.56 ± 37

ALP = alkaline phosphatase, ALT = alanine transaminase, AST = aspartate aminotransferase, RI = reference intervals

**Table 2:** Haematology of the male African pygmy hedgehog

Parameter	SI units	Value	RI (Ivey and Carpenter 2020)
Haemoglobin	g/l	107	120 ± 28
Haematocrit	%	34	36 ± 7
Erythrocytes	10^12^/l	5.12	6 ± 2
Leukocytes	10^9^/l	12.53	11 ± 6
Thrombocytes	10^9^/l	218	226 ± 108
Lymphocytes	10^9^/l	2.57	4.0 ± 2.2
Monocyte	10^9^/l	1.04	0.3 ± 0.3
Neutrophils	10^9^/l	7.94	5.1 ± 5.2
Basophils	10^9^/l	0.44	0.4 ± 0.3
Eosinophils	10^9^/l	0.54	1.2 ± 0.9

RI = reference intervals

**Figure 2:**
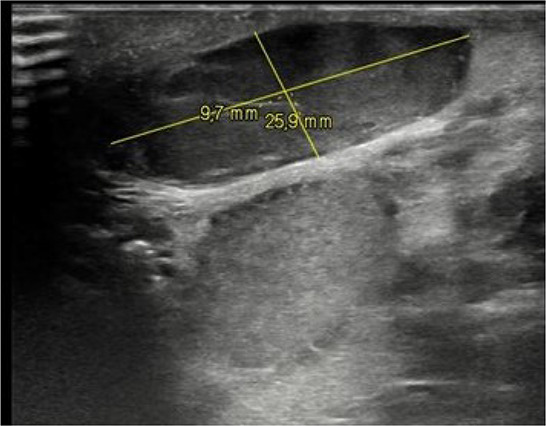
Ultrasonographic appearance of a mass inside the penile sheath of the 3-year-old African pygmy hedgehog Same echogenicity as the polyp-shaped tumour-like mass protruding from the preputium

**Figure 3:**
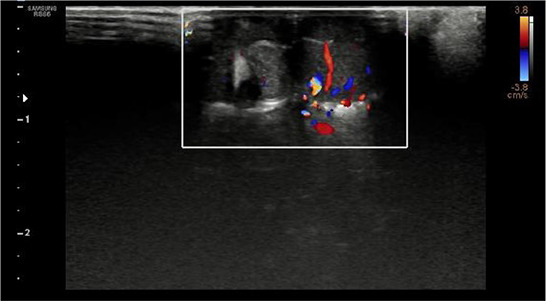
Doppler ultrasound revealing the major vascularisation of the observed penile mass of the 3-year-old African pygmy hedgehog

Ultrasonography with a linear probe (LA4–18B, frequency 4–18 MHz; Samsung RS85 prestige; Samsung Medison Co., Ltd., Seoul, Republic of Korea) revealed a mass (2.5 × 0.9 cm) inside the penile sheath, with the same echogenicity as the polyp-shaped mass protruding outside (Figure 2). Examination with Doppler ultrasonography (linear probe LA4–18B, frequency 4–18 MHz; Samsung RS85 prestige; Samsung Medison Co., Ltd., Seoul, Republic of Korea) revealed major mass vascularisation (Figure 3). Both testicles were visualised, the right testicle (1.3 × 0.5 cm) was situated in the pouch close to the anus, while the left testicle (1.8 × 1.3 cm) was found in the abdominal cavity. The ultrasound did not reveal any other changes in the abdominal cavity.

**Figure 4:**
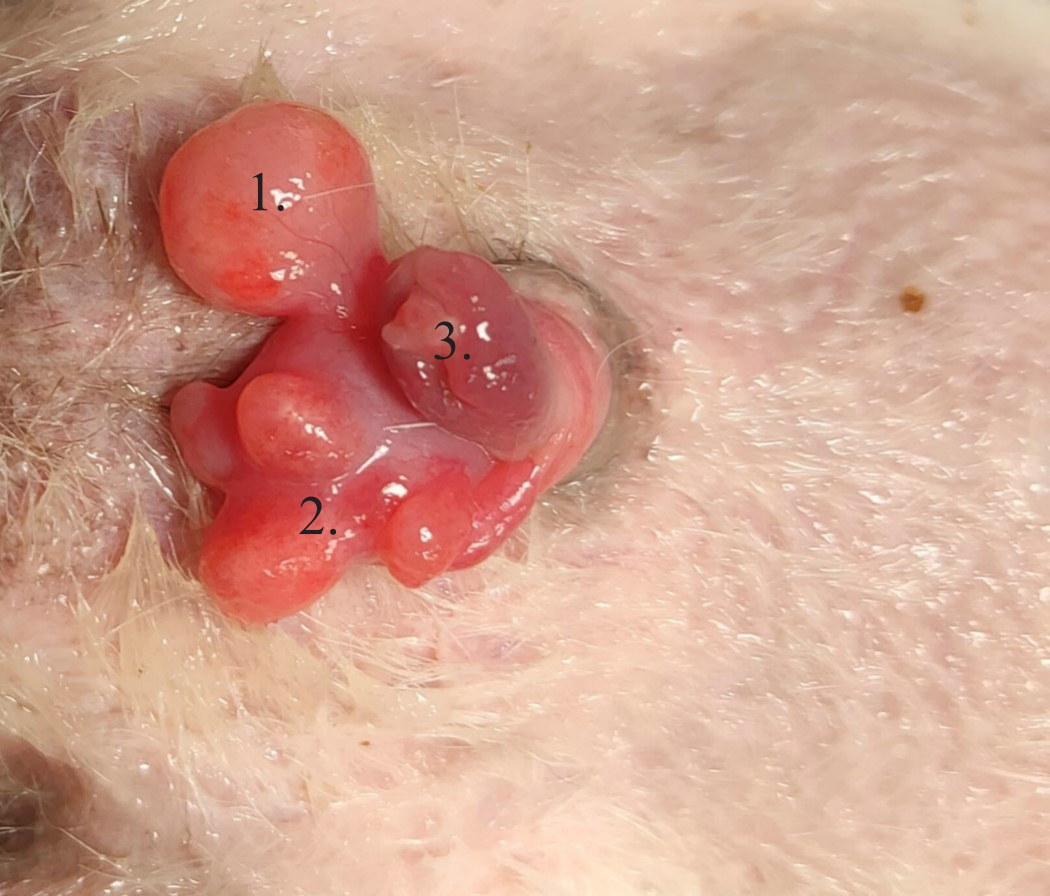
Exposed penis from the penile sheath with the polyp and lobular tumour in the 3-year-old African pygmy hedgehog Polyp-shaped mass (1) protruding from the preputium. Visualised lobular mass (2) exposed from the penile sheath. Glans penis (3)

After a topical anaesthetic gel with trimecaine (Mesocain; Zentiva International a.s., Bratislava, Slovak Republic) was applied to the preputium, it was possible to expose the penis and attached masses out of the penile sheath (Figure 4).

**Figure 5:**
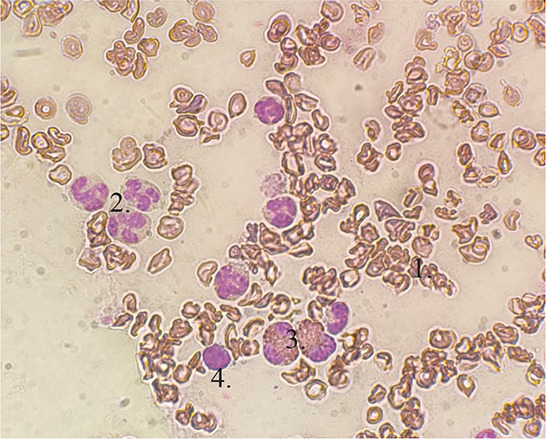
Impression smear of the polyp mass on the penis of the 3-year-old African pygmy hedgehog Erythrocytes (1), neutrophils (2), eosinophils (3), small lymphocyte (4). H&E, magnification × 40

Using the same anaesthetic protocol with 3% isoflurane (Aerrane 100% 250 ml; Baxter S.A.Bd., Lessines, Belgium) and an O_2_ flow of 2 l/min, the polyp–shaped mass was ligated using a resorbable poly-filament suture (PGA synthetic absorbable poly-filament, PGA 4-0; Resorba Medical GmbH, Nürnberg, Germany) and removed without any complications and significant bleeding. Cytology of an impression smear from the lesion was performed and the mass was sent for a histopathological examination. The mass was fixed in a 10% formol solution prior to the examination. The cytology findings included mostly red blood cells, and leucocytes (Figure 5). Eosinophils, monocytes, and neutrophils were all observed without any signs of bacterial infection. The examined stromal cells appeared normal.

Shortly after the patient woke up, it was able to urinate. The observed urine was without any macroscopic signs of blood. The penis remained prolapsed due to the inability to return the structures inside of the preputium. The patient was given supportive therapy: Fluids with amino acids and vitamins were administered subcutaneously – Hartmann solution (B Braun, Melsungen, Germany) and Duphalyte (Zoetis Manufacturing Research Spain SL, Girona, Spain) in a ratio of 5 : 1 (10 ml *pro toto* s.c. q12 h). Analgesia consisted of meloxicam (0.2 mg/kg s.c. q12 h; Melovem 5 mg/ml; Dopharma research B.B., Raamsdonksveer, The Netherlands) and local anaesthetic trimecaine (Mesocain gel q24 h; Zentiva International a.s., Bratislava, Slovak Republic) that was applied on the prolapsed penis. Other medications included antiemetic prokinetic maropitant (1 mg/kg s.c. q24 h; Cerenia 10 mg/ml; Zoetis Belgium SA, Louvain-la-Neuve, Belgium) and the H2 blocker famotidine (0.4 mg/kg i.m. q12 h; Quamatel 20 mg/5 ml; Gedeon Richter Plc., Budapest, Hungary). Due to monocytosis present and the results of the impression smear, the patient received antibiotic therapy consisted of marbofloxacin (10 mg/kg i.m. q24 h; Marbocyl 100 mg/ml; Vétoquinol, Lure, France).

**Figure 6:**
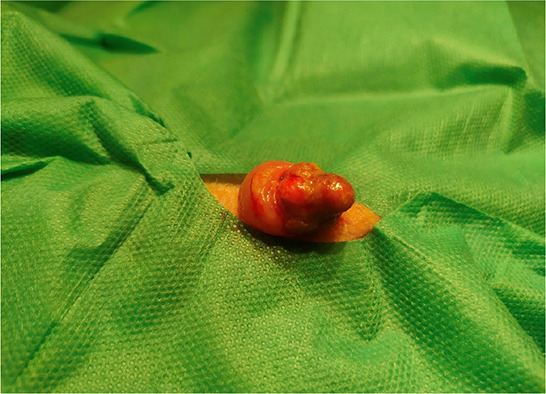
Prepared surgical field of the male 3-year-old African pygmy hedgehog The penile mucous membrane is swollen with signs of automutilation and bleeding. The urethral opening is not visible due to the oedema and the presence of the multilobular mass (myxofibrosarcoma of the penis)

**Figure 7:**
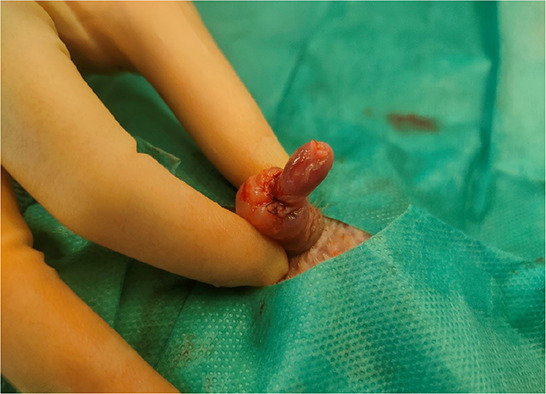
Glans penis and swollen preputial mucosa of the penis in the 3-year-old African pygmy hedgehog After removing the lobulated mass, it was possible to visualise the dorsal part of the penile glans. Urethral process with two typical needle-shaped structures

**Figure 8:**
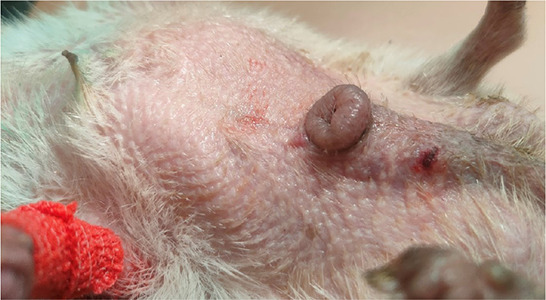
African pygmy hedgehog, 3-year-old Second day post-surgery, it was possible to return the prolapsed penis as the swelling decreased. Note, the ongoing swelling of the lower abdomen after the penis was returned

During the following three days, the prolapse was cleaned daily, with an effort to return the penis to the penile sheath, but without any positive outcome. From the fourth day, the mucous membrane started to be more swollen and fragile, with multiple bleedings on the surface, otherwise, the observed structures remained the same size. The patient was able to urinate; however, there was a small decrease in the food intake and activity. The owner agreed to proceed to surgery prior to receiving the results of the histopathology report obtained from the polyp-shaped mass (Figure 6). Premedication consisted of buprenorphine (0.03 mg/kg s.c.; Bupaq 0.3 mg/ml; Richter Pharma AG, Wels, Austria) after which the patient was induced to inhalation anaesthesia using mask induction, with 5% isoflurane with a flow of O_2_ 2 l/min. During the procedure, the patient was maintained at 3% with a mask. The mass was ligated using a single suture with resorbable poly-filament material (PGA 4-0; Resorba Medical GmbH, Nürnberg, Germany) and was successfully removed without any severe bleeding. Due to additional oedema of the preputium and the fascia of the dorsal side of the penis, the prolapse could not be returned (Figure 7). The second-day post-surgery the oedema decreased and it was possible to return the prolapsed penis back to the prepuce (Figure 8). During the following days, the swelling of the preputium continued to decrease, and the patient was discharged from the hospital. The at-home medication included meloxicam (0.2 mg/kg p.o. q12 h; Meloxicam 1.5 mg/ml; Bioveta, a.s., Ivanovice na Hané, Czech Republic) and marbofloxacin (10 mg/kg p.o. q24 h; Quiflox 5 mg/tbl; Krka, d.d, Novo mesto, Slovenia).

**Figure 9:**
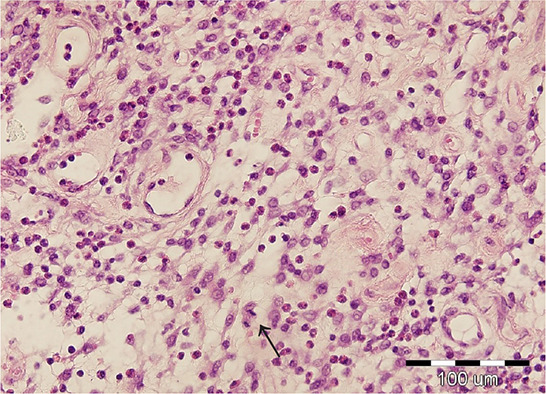
Myxofibrosarcoma of the penis in the 3-year-old African pygmy hedgehog The mass consists of spindle-shaped to oval cells with mild atypia (anisocytosis, anisokaryosis, binucleation, prominent nucleoli). Rare mitotic figures were observed (arrow). H&E, magnification × 400

While the histopathological examination of the polyp-like mass revealed a chronic active inflammatory reaction, the histopathological examination of the multilobular mass revealed a myxofibrosarcoma. The tumorous tissue of the multilobular mass was composed of spindle–shaped cells, irregularly oval or polygonal in some places. Focal tumour cells with a myxoid differentiation were observed in the greater part of the tumour. Mild cellular atypia like anisocytosis, anisokaryosis, prominent nucleoli, multinucleated cells, and rare mitotic figures were observed (Figure 9). The stroma was made up of sparse fibrous tissue. The surface epithelium was hyperplastic with ulcerations and necrosis. The owner did not consent to us performing a follow-up immunohistochemical examination of the tumour.

The histopathological examination did not confirm whether there were any clear margins of the resected tumour.

Two weeks post-surgery, the penile swelling completely disappeared, and the patient was active and healthy, which was confirmed by a phone consultation. After two weeks, the patient was lost to any more follow up.

## DISCUSSION

To date, limited information about the male reproductive tract of the African pygmy hedgehog (*Atelerix albiventris*) is available. The most common disease associated with the male reproductive tract recorded is posthitis of the preputium, due to the presence of substrate or foreign materials. This could turn into a prolapsed and injured penis (Ivey and Carpenter 2020). A retrospective study of disease incidence in African pygmy hedgehogs mentioned one case of hyperplasia of seminal vesicles without any further description (Okada et al. 2018). This pathology was later described in a case of gastrointestinal obstruction secondary to seminal vesicle cystic hyperplasia (Wooten and Snider 2022). The only described case of a penile tumour in African pygmy hedgehog was a myxoma of the penis, where the patient (intact male, 3.5-years-old, 648 g) was presented with a solid mass in the preputium. In their case, after a complete excision of the myxoma, there were no clinical abnormalities or metastasis in the lymph nodes observed post-operatively up to 100 days (Takami et al. 2017). A myxoma is a benign tumour of the connective tissue with simple histopathological features. Compared to the myxoma, myxofibrosarcoma is presented with more histological heterogeneity (Martin-Carreras et al. 2019). They are classified as soft tissue sarcomas, originating from fibroblasts, composed of a myxoid matrix with the presence of mucopolysaccharides (Iwaki et al. 2019).

There is minimal information available about canine and feline myxofibrosarcomas. Soft tissue sarcomas are present in around 15% of dogs and only 7% in cats (Liptak and Christensen 2020). These statistics include multiple benign (fibrosarcoma, leiomyosarcoma, rhabdomyosarcoma, etc.), and malignant tumours (mesenchymoma, spindle cell carcinoma, myxofibrosarcoma, etc.) (Liptak and Christensen 2020). In humans, myxofibrosarcomas present in about 5% of all soft tissue sarcomas, with the most common locations being at their extremities (Iwaki et al. 2019). In dogs, they are usually located in the trunk and limbs. However, there are reports of myxosarcomas of the internal organs as well, such as the spleen, heart, or lungs (Iwaki et al. 2019). In dogs, sarcomas are characterised as a locally aggressive tumour, with recurrence rates from 16–57% (Iwaki et al. 2019).

The treatment of myxofibrosarcomas in veterinary medicine is yet not well described. A study consisting of 32 dogs presented multiple options and results for the therapy of myxosarcomas (Iwaki et al. 2019). All the dogs from the study underwent surgical resections. Depending on the patient, this was the only treatment, or it was followed with radiation or chemotherapy. The used chemotherapeutics included cyclophosphamide, chlorambucil, doxorubicin, toceranib, masitinib, and rapamycin. In dogs with a low mitotic index, the local recurrence was low, with a median time of 115 days (Iwaki et al. 2019).

As for the use of chemotherapy in African Pygmy hedgehogs, there are no established protocols. The use of chemotherapeutical substances is usually associated with eosinophilic leukaemia, where a combination of prednisone and cytarabine is used (Koizumi et al. 2020). In a more recent case, an oral spindle carcinoma was treated with carboplatin (Lacqua et al. 2022). The patient stopped to respond to the medication after two months and was then switched to lomustine. This decreased the size of the mass for another six months, after which the mass reappeared and the patient was treated with radiotherapy. After the radiotherapy, the patient developed further complications and, after confirming the presence of metastases, the patient was humanely euthanised (Lacqua et al. 2022).

In the presented case, the first observed polyp-shaped mass with active inflammatory and a reactive fibroblastic reaction (inflammatory polyp) was detected in the connective stroma. The histopathological examination of the second, multilobular mass, revealed a myxofibrosarcoma (tumour of the lower degree of malignancy – soft tissue spindle cell sarcoma, myxofibrosarcoma grade 1). The ultrasound did not reveal any potential metastases in the abdominal cavity of the patient. Two weeks post-surgery, the patient did not show any clinical signs of a disease, which was confirmed by the owner by phone consultation. Considering the lifespan of African pygmy hedgehogs, the surgical removal of a myxofibrosarcoma with clear margins should prevent the risk of recurrence. In this case, it was not possible to contact the owner again after 6 months, and the patient was lost to any further follow-up.
